# MRI-guided intrathecal transplantation of hydrogel-embedded glial progenitors in large animals

**DOI:** 10.1038/s41598-018-34723-x

**Published:** 2018-11-07

**Authors:** Izabela Malysz-Cymborska, Dominika Golubczyk, Lukasz Kalkowski, Adam Burczyk, Miroslaw Janowski, Piotr Holak, Katarzyna Olbrych, Joanna Sanford, Kalina Stachowiak, Kamila Milewska, Przemysław Gorecki, Zbigniew Adamiak, Wojciech Maksymowicz, Piotr Walczak

**Affiliations:** 1 Department of Neurosurgery, School of Medicine, Collegium Medicum, University of Warmia and Mazury, Olsztyn, 10-719 Poland; 2 Faculty of Biology and Biotechnology, University of Warmia and Mazury, Olsztyn, 10-719 Poland; 3 NeuroRepair Department, Mossakowski Medical Research Centre, Polish Academy of Sciences, Warsaw, 02-106 Poland; 4 Institute for Cell Engineering, Cellular Imaging Section, The Johns Hopkins University School of Medicine, Baltimore, MD 21205 USA; 5 Division Russell H. Morgan Dept. of Radiology and Radiological Science, The Johns Hopkins University School of Medicine, Baltimore, MD 21205 USA; 6 Department of Surgery and Roentgenology with the Clinic, Faculty of Veterinary Medicine, University of Warmia and Mazury, Olsztyn, 10-719 Poland; 7 Department of Morphological Sciences, University of Life Sciences – SGGW, 02-787 Warsaw, Poland; 8 Vet Regen, Warsaw, 04-844 Poland; 9 Department of Mathematics and Informatics, University of Warmia and Mazury, Olsztyn, 10-719 Poland

## Abstract

Disseminated diseases of the central nervous system such as amyotrophic lateral sclerosis (ALS) require that therapeutic agents are delivered and distributed broadly. Intrathecal route is attractive in that respect, but to date there was no methodology available allowing for optimization of this technique to assure safety and efficacy in a clinically relevant setting. Here, we report on interventional, MRI-guided approach for delivery of hydrogel-embedded glial progenitor cells facilitating cell placement over extended surface of the spinal cord in pigs and in naturally occurring ALS-like disease in dogs. Glial progenitors used as therapeutic agent were embedded in injectable hyaluronic acid-based hydrogel to support their survival and prevent sedimentation or removal. Intrathecal space was reached through lumbar puncture and the catheter was advanced under X-ray guidance to the cervical part of the spine. Animals were then transferred to MRI suite for MRI-guided injection. Interventional and follow-up MRI as well as histopathology demonstrated successful and predictable placement of embedded cells and safety of the procedure.

## Introduction

The concept of stem cell-based therapy for neurological disorders is few decades old and while some progress has been made in this area, the efficacy of treatment is rather low. Multifocal or disseminated diseases such as multiple sclerosis (MS) or amyotrophic lateral sclerosis (ALS) pose a particular challenge due to disseminated character and the subsequent need for cell delivery to the extensive areas of the brain and spinal cord. Stereotaxic intraparenchymal injection, which is currently a dominating technique for cell delivery^1^ seems to be not feasible for disseminated disease as dozens or even hundreds of injection sites would be required to cover brain volume that is meaningful for achieving therapeutic effect.

Cerebrospinal fluid (CSF) space surrounding the central nervous system (CNS) offers unique gateway for cell delivery. The CSF circulation allows spreading of transplanted cells in the CNS without surgical intervention. The CSF and particularly intrathecal space can be easily and minimally invasively accessed *via* lumbar puncture^2^. Indeed, this route has been already exploited for cell delivery, including preclinical^3^ and clinical applications^4^. However, the unpredictability of cell biodistribution within fluid-filled space with a possibility for sedimentation or their removal hampers the progress in the field. These challenges can be addressed by embedding therapeutic cells in injectable hydrogel as a biomaterial which provide a support for the cells and would assure secure and precise placement at the spinal cord surface. Hyaluronic acid (HA) based hydrogel was shown to support cell survival after transplantation and its biomechanical properties and gelation dynamics facilitates injectability^5^. Assumptions of this approach that formed basis for this study were that stem cells embedded in the injectable hydrogel would remain localized in the place of the injection and that the hydrogel would support cell survival and migration into spinal cord parenchyma and that the gel deposit would not trigger inflammation or block circulation of the CSF.

The precision medicine including cell delivery process is drawing increasing attention. Image-guidance may perfectly address this requirement and facilitate precise, minimally invasive deployment of transplanted cells within the fluid-filled spaces. The scaffolding of cells within hydrogel prevents the uncontrollable biodistribution within CSF reservoirs and provide a tool to control the process of cell infusion. Here, we took advantage of our previously developed dynamic interventional MRI based on the strong T2* contrast of iron oxide nanoparticles^6^. Labeling of the hydrogel with iron oxide nanoparticles enables accurate and dynamic visualization of the hydrogel placement during its infusion in real-time using MRI, while obviating incorporation of label into cells. Such instant feedback about the placement of the gel provides opportunity for adjusting and optimizing its biodistribution.

Neural progenitor cells as they are multipotent are widely used in preclinical studies for CNS injuries and disorders. Depending on the needs and desired therapeutic mechanism stem cells at different level of differentiation can be used as transplantation material. Less differentiated cells such as neural stem cells (NSCs) have broader differentiation capacity but at the price of difficulty in controlling downstream phenotypes. More committed progenitor cells such as neural (NRP) or glial (GRP) precursors are more restricted in their differentiation repertoire but at higher certainty about the phenotype of their mature products^7^. Rat GRPs when were transplanted into adult spinal cord survived for at least 6 weeks and differentiated into astrocytes and oligodendrocytes. Moreover, cells exhibited robust engraftment and migration along white but no gray matter reaching more than 15 mm during 6 weeks^8^. Similarly, human GRPs, transplanted into injured rat spinal cords survived, migrated through the spinal cord tissue, and exerted restorative effects including preserved electrophysiological conduction across the spinal cord^9,10^. While rodents are prevalently used for modeling neurodegenerative diseases, like ALS, they often lack clinical relevance. There is a growing consensus that companion animals are a unique resource for late stage preclinical therapy studies. Naturally occurring diseases such as degenerative myelopathy (DM) in dogs closely resemble human ALS. Therefore, in this study we took advantage of that opportunity and based on previous success with cell transplantation in small animals we investigated safety and feasibility of MRI-guided intrathecal transplantation of glial restricted progenitor cells (GRPs) embedded in the HA hydrogel in dogs suffering from DM before eventual introducing to the clinic.

## Results

### Effect of hydrogel on GRPs survival

**Figure 1:**
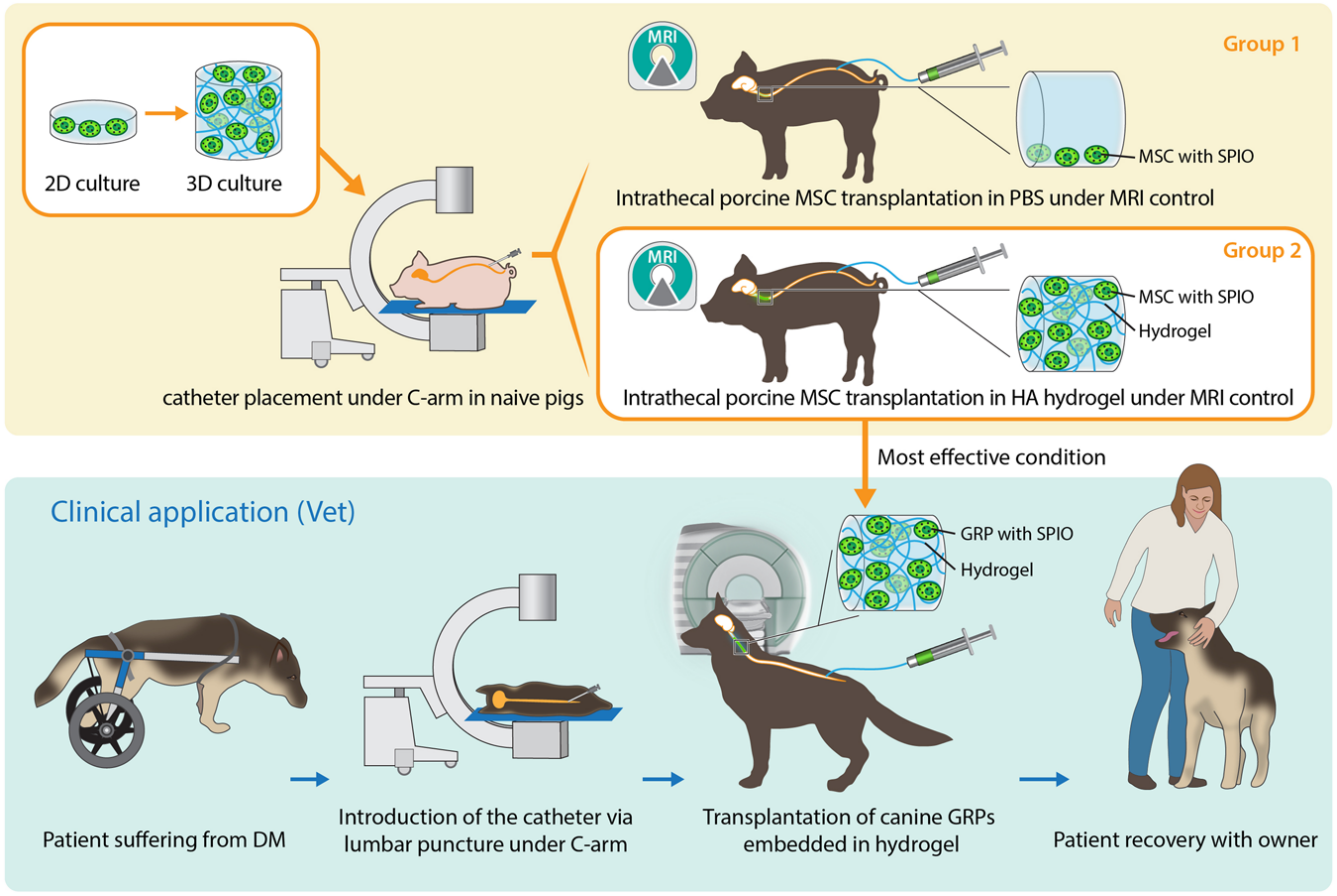
Experimental design. Figure illustrated by I-Hsun Wu.

**Figure 2:**
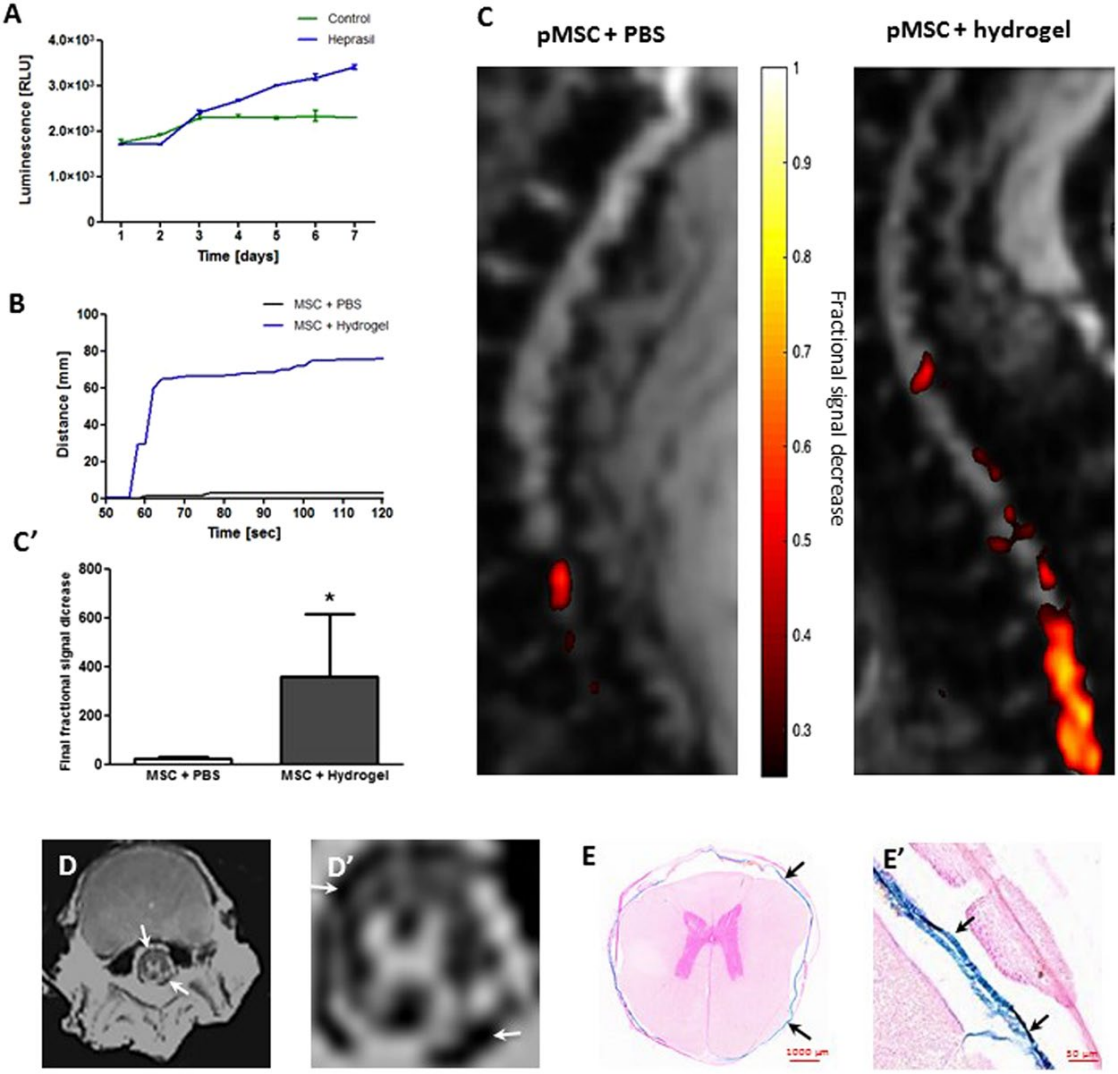
Effect of embedding stem cells in hydrogel for intrathecal transplantation in pigs: (**A**) Influence of hydrogel on mouse GRPs viability, (**B**) Distance of spinal cord tissue of the pig, covered by the pBMMSC in PBS and hydrogel, (**C**,**C**’) Real-time MRI monitored pBMMSC transplantation in pig, (**D**,**D**’) *Ex vivo* MRI of porcine spinal cords after transplantation of pBMMSC embedded in hydrogel, (**E**,**E**’) Prussian blue staining of porcine spinal cords after transplantation of pBMMSC embedded in hydrogel (arrows indicate transplanted cells-hydrogel composites).

GRPs were cultured either as a monolayer or were embedded in a heparin supplemented HA hydrogel (Heprasil®) and were incubated for seven days with daily measurements of their survival and proliferation with bioluminescence. Hydrogel embedded cells survived well and proliferated at the rate that was higher compared to the control (Figs 1, 2A; P < 0.05).

### Comparison of biodistribution of pBMMSCs suspended in PBS vs. embedded in hydrogel during transplantation in pig using Real-time MRI

Dynamic imaging during injection procedure enabled observation of pBMMSC labeled with SPION as they gradually distributed through the CSF-filled intrathecal space. The hypointense signal was visible in the area of the catheter tip expanding rostrally and caudally in the thoracic part of the spinal cord. The hypointense signal of pBMMSCs suspended in PBS was much lower in comparison to signal of cells embedded in hydrogel (Figs 1, 2B, C; P < 0.05). This is likely due to the dispersion of cells within CSF with their concentration per imaging voxel below detection limit. The maximum distance of the spinal cord tissue with detectable signal for cells in PBS was only at 0.25 cm, whereas distance for cell-hydrogel composites was for up to 12.0 cm. The total number of hypointense pixels counted as a final fractional signal decrease was much higher in the pBMMSC group embedded in hydrogel (Figs 1, 2C’; P < 0.05).

### *Ex vivo* MRI and histology

The confirmation of accurate intrathecal transplantation and placement of SPIO-labeled HA-based hydrogel in pig was conducted *post mortem* using MRI. The *ex vivo* MRI T2*weighted scans of tissue explants showed hypointense regions in the thoracic section (Th10-Th12) of the spinal cord (Fig. 2D, D’). The hypointensity area fully surrounded the spinal cord (Fig. 2D’). Additionally, histological preparation and Prussian blue staining detected iron nanoparticles in the same location surrounding the spinal cord tissue (Fig. 2E, E’).

### Feasibility of Real-time MRI monitored cGRPs transplantation in dogs with degenerative myelopathy

**Figure 3:**
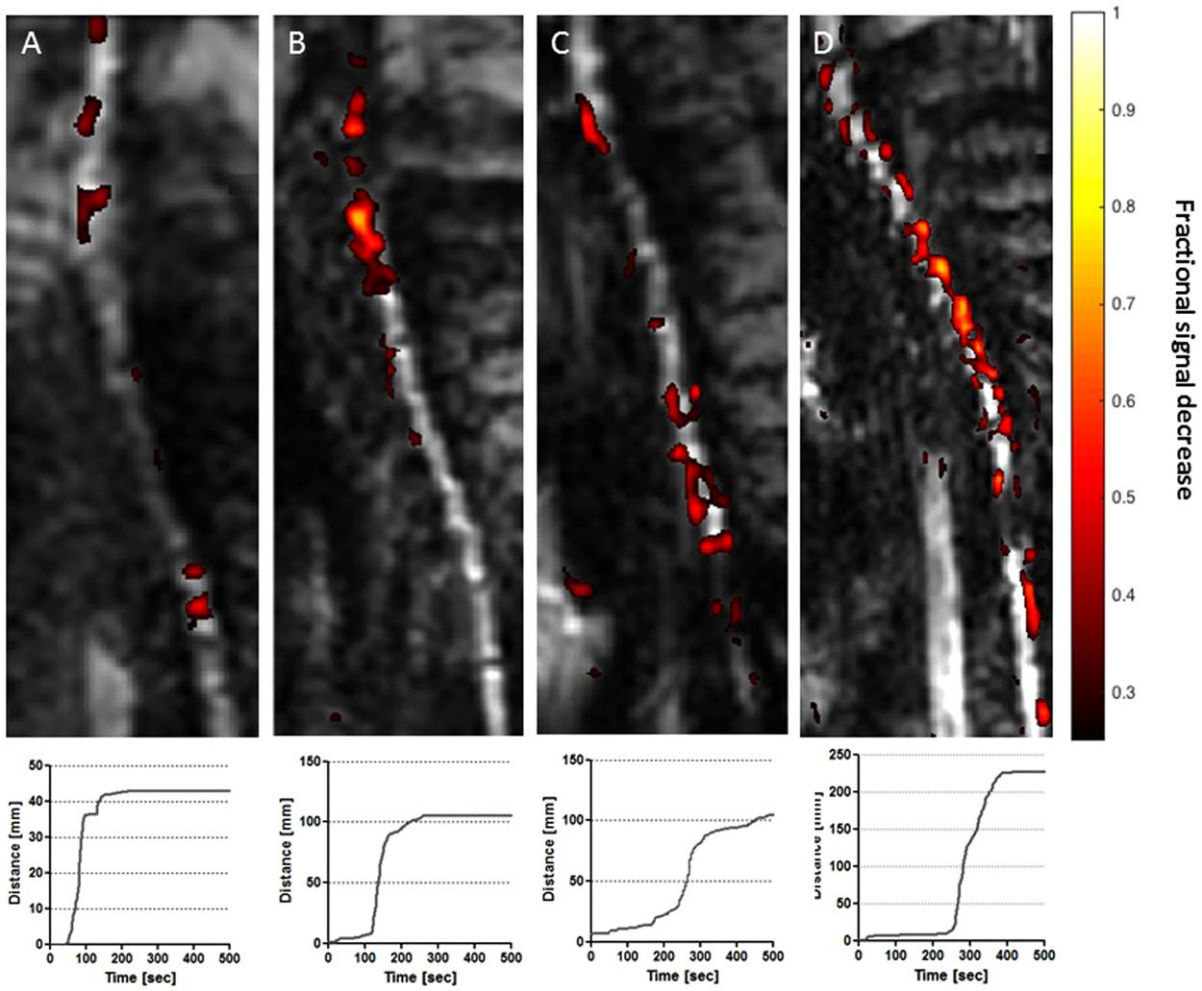
MRI-guided intrathecal transplantation of SPION canine GRPs embedded in HA-based hydrogel.

Pig studies provided evidence about feasibility and were used to optimize MRI-guided intrathecal injection procedure. With these critical data we were able to proceed to therapeutic use of canine glial progenitors (cGRPs) in dogs suffering from degenerative myelopathy. Due to the degeneration of the lumbar spine, often occurring in dogs over 7 years of age, intrathecal injection is not easy, which in this case we were able to successfully perform. The cGRPs, labeled with SPION were embedded in Heprasil® (supplemented with SPION) prior to crosslinking of the hydrogel. Initiation of the injection 2 min after mixing hydrogel components (Heprasil and cross-linker) was established as optimal and enabled passage of the hydrogel through the catheter and desired gel formation in CSF. Dynamic imaging during hydrogel infusion revealed hypointensity in the area of the catheter tip expanding rostrally and caudally in the thoracic part of the spinal cord. The rostral-caudal distance, covered by cells-hydrogel formulation ranged between 4.2 and 25 cm (Figs 1 and 3A–D). The hydrogel spreading in the CSF and persistence in the intrathecal space was visible under MRI throughout the entire imaging timeframe (9 mins; Figs 1, 3A–D). Analysis of signal intensity plots obtained during the cGRP transplantation revealed that the hypointense regions increased over time and reached plateau without subsequent reduction confirming that cells-hydrogel composites remained in place after transplantation (Figs 1, 3A–D).

### Histological detection of transplanted hydrogel-cell composites

**Figure 4:**
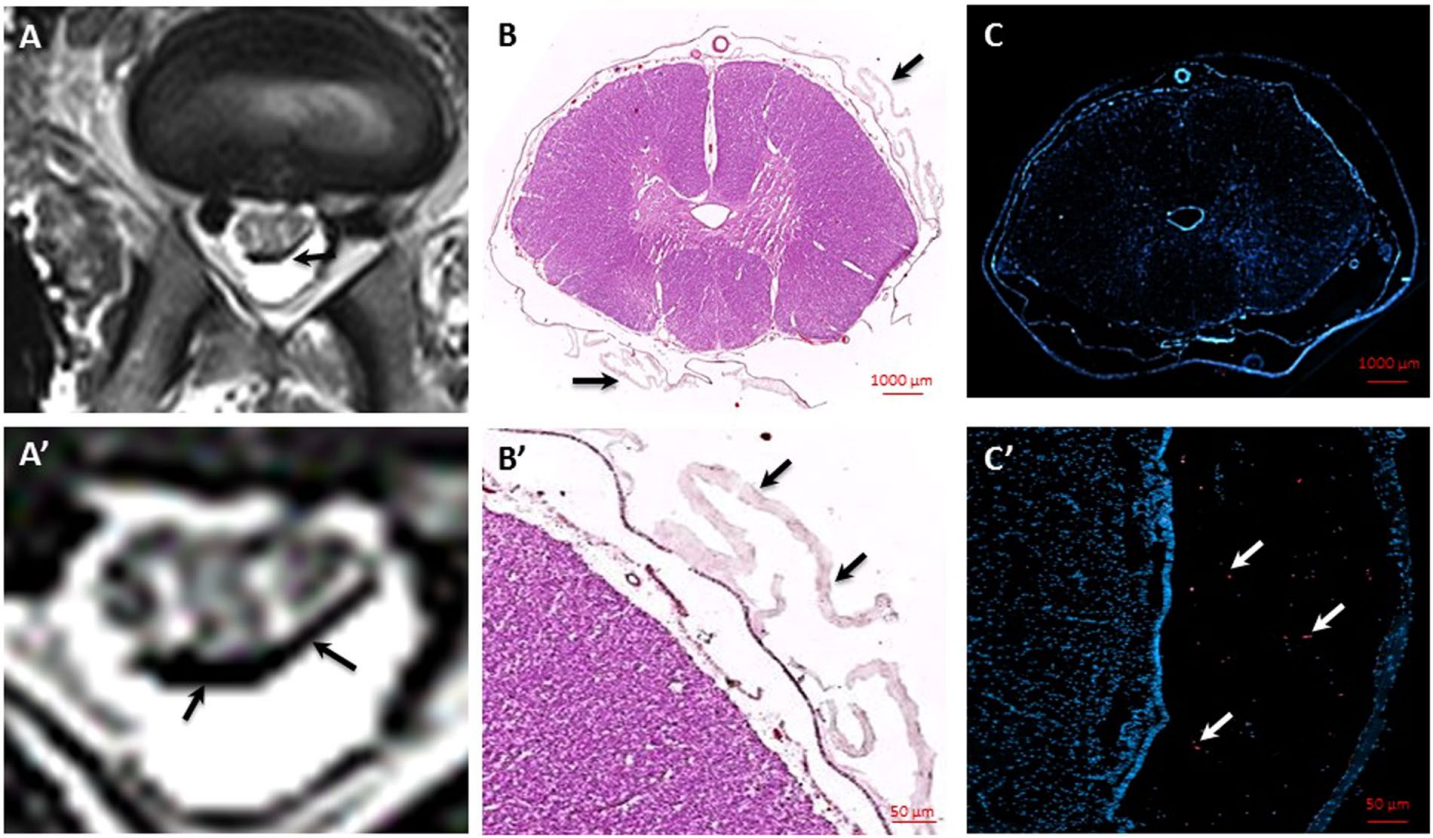
Histopathological analysis of spinal cords of dogs after transplantation of cGRPs embeded in HA-based hydrogel. (**A**, **A**’) *Ex vivo* MRI, (**B**, **B**’) HE staining (10x and 40x magnification), (**C**, **C**’) DAPI staining (10x and 40x magnification). Arrows indicate transplanted cells-hydrogel composites.

*Ex vivo* T2*-weighted MRI scans of spinal cord tissue of dogs, that succumbed to the disease demonstrated distribution of hypointensities mirroring those shown during interventional cGRP transplantation procedure (Fig. 4A, A’). Histopathology further confirmed imaging findings as HE staining showed hydrogel surrounding the spinal cord tissue in hypointensity regions visible on MRI (Fig. 4B-B’). Fluorescence microscopy for rhodamine-tagged SPIO in labeled cGRPs and hydrogel, facilitated detection of cells in the hydrogel (Fig. 4C-C’).

### Safety of intrathecal transplantation of hydrogel-embedded GRPs

**Figure 5:**
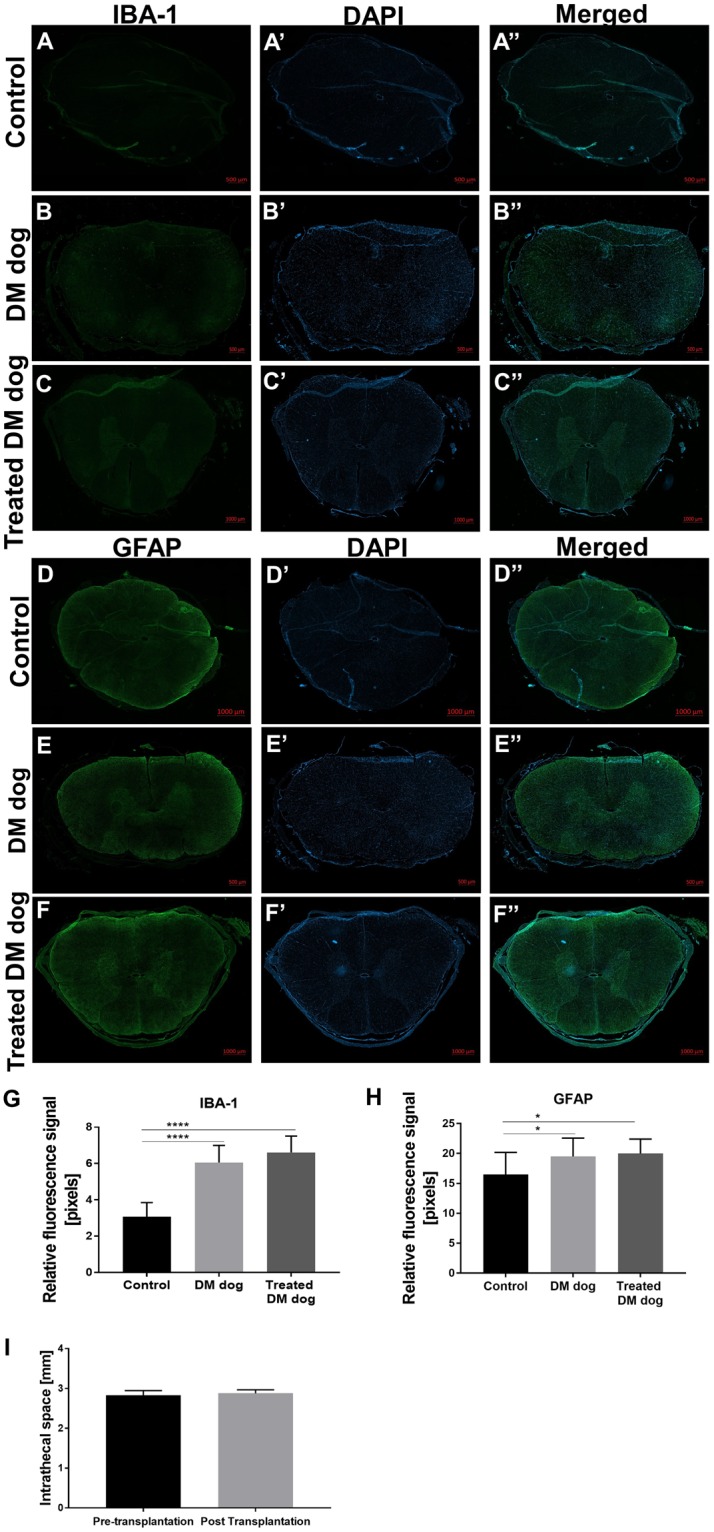
Safety of MRI-guided intrathecal transplantation of SPION canine GRPs embedded in HA-based hydrogel. (**A–H**) Immunofluorescence staining of Iba-1 (**A-C**”, **G**) and GFAP (**D**-**F**”, **H**) in spinal cord tissue of DM dogs, (**I**) Evaluation of intrathecal space dimensions visible on the MRI before versus post-transplantation.

The IBA-1 immunoreactivity for activated microglia/macrophages was used to detect inflammatory response in the vicinity of the graft. Historical samples of untreated dog spinal cord tissue (Courtesy of Dr. Joan Coates from the University of Missouri) both naive and suffering from DM served as baseline control. There was significant elevation of IBA-1 immunoreactivity in untreated DM dogs (Fig. 5A-C’’, G; P < 0.0001), compared to control. In cGRP-treated dogs IBA-1 expression was at the level of untreated DM dogs (Fig. 5A-C’’, G; P < 0.0001). Reactive astrocytosis is another hallmark of neuroinflammation and it was measured by immunostaining for the glial fibrillary acidic protein–GFAP. Higher astrocytosis was detected in DM dogs, both in white and grey matter of spinal cord tissue of treated and untreated animals compared with the control dog (Fig. 5D-F’’, H; P < 0.05).

Injection of biomaterials into the intrathecal space introduces the risk of blocking CSF circulation and resulting accumulation. To address this, we measured the intrathecal space visible on the MRI before versus post-transplantation. There was no expansion of intrathecal space at any level when measured before and after transplantation (Fig. 5I).

## Discussion

Intrathecal injections are widely used in clinic for drug delivery. This made it a promising conceptual window to therapy in disorders with widely disseminated lesions. The intrathecal technique of cell injection was studied in several animal models^11,12^. This route for stem cell delivery *via* lumbar puncture was shown to be safe and feasible even with high number of transplanted cells^1,13,14^. For example, MSC transplantation into CSF in patients suffering from ALS was safe as no side effects were reported; however, only a modest immunomodulatory effect have been observed^4^. Without means for monitoring biodistribution of cells after injection was uncertain and indeed the authors indicated suboptimal biodistribution or sedimentation as a reason for the lack of therapeutic efficacy.

Biomaterials and specifically injectable hydrogels have potential to be the key reagents for success of intrathecal delivery and targeted cell placement. Hyaluronic acid-based hydrogels have positive effect on attachment, migration and differentiation of variety types of stem cells^5,15,16^. In the current study we used the biomaterials to achieve two primary effects: support survival of the embedded cells and to control precision of cell biodistribution. Indeed, recently in the current study, we demonstrated that embedding of GRPs in HA hydrogels results in their improved viability as measured by *in vitro* luciferase assay. *In vivo* experiments in pigs with intrathecal injection of cells labeled with iron oxide nanoparticles for detection in MRI, demonstrated that when cells are suspended in PBS (without the hydrogel) the cells rapidly disperse, quickly becoming undetectable by MRI. In contrast, embedding labeled cells in the hydrogel prevented their dispersion and resulted in accurate placement.

Image-guided delivery is crucial for assuring proper biodistribution of the hydrogel in the CSF. With real-time MRI monitoring during transplantation it is possible to observe biodistribution of SPIO-labeled cell-hydrogel composites in the CSF. Thanks to the continuous MRI visualization, this approach facilitates instant reaction- adjusting placement of the catheter or to stop the injection. Speed of injection is another factor that could be used to optimize material distribution as it was earlier investigated^17^.

In canine degenerative myelopathy neurodegenerative processes are prominent in thoracic region of the spinal cord thus for the veterinary clinical application of our cell delivery strategy we utilized MRI-guided transplantation of GRPs embedded in HA hydrogels with the goal of demonstrating safety of the procedure and feasibility of cell targeting selectively to the thoracic spinal cord. Interventional MRI indeed successfully demonstrated placement of the cell/biomaterial and it was instrumental in assuring biodistribution of the material at desired sites.

Dynamic imaging after completed injection showed signal stability indicating cells remained at side of injection within intrathecal space. Moreover, our histopathological study of spinal cord of dogs that succumb to the DM two months post transplantation revealed presence of the hydrogel in the T7-T9 regions, that was analogous to real-time *in vivo* and *post mortem ex vivo* MRI. In histopathology for Prussian blue staining hydrogel was clearly visible as a blue layer fully surrounding the spinal cord tissue. In contrast to intraparenchymal cell injection this broad distribution is great advantage as implanted cells have contact with large surface of the spinal cord. This also is an excellent starting point for studying and promoting migration of cells from the hydrogel into the spinal cord parenchyma.

Important aspect when performing biomaterial injections into the CSF is the risk of interfering with CSF circulation which is potentially dangerous for the patient. Several studies indicated safety and feasibility of intrathecal hydrogel injection in the rodent model^18,19^. To address this concern in large animals, we performed measurements of intrathecal space based on anatomical T2 weighted MRI and we did not observe any evidence of CSF circulation block. To conclude, our study showed for the first time that intrathecal transplantation of cells embedded in hydrogel into CSF of large animals is safe and feasible. Interventional MRI is instrumental in assuring precision of cell placement. This brings hope for patients suffering for ALS and many other neurological disorders.

## Materials and Methods

### Isolation of porcine mesenchymal stem cells (pMSC)

The bone marrow (BM) was aspirated from juvenile pig iliac crest using a syringe containing 10 ml PBS with 200 µl heparin (Polfa, Warsaw, Poland). The bone marrow was diluted with PBS (Gibco, Gaithersburg, MD, USA) in a ratio of 1:2. Next, the mixture was layered on a Ficoll- Paque Plus (Sigma Aldrich, Germany) in a 2:1 ratio and centrifuged at 1300 rpm at room temperature (RT) for 25 min. A ring of mononuclear cells was collected into a new tube with 20 ml of PBS and centrifuged at 1500 rpm for 10 min in RT. Next the pellet of cells was washed 2 times with PBS. Cells were suspended in the BMMSC medium (Gibco) and plated on 25 ml flasks in 37 °C in humified incubator with 5% CO_2_. Cultured cells were maintained for 15–20 days (2–3 passages), harvested with Accutase (Gibco), cryopreserved in freezing medium (DMEM with 40%FBS + 5%DMSO; Gibco; Sigma Aldrich), and stored in vapor phase liquid nitrogen.

### Isolation of canine glial restricted progenitors (cGRP)

Brains and spinal cords were dissected from canine fetuses between E32–37. Briefly, the tissue was incubated in pre-warmed TrypLE Express (Gibco) with 10 mg/ml DNase-1 (A&A Biotechnology, Gdansk, Poland) for 10–12 mins, gently triturated and incubated in 37 °C for 10 min. Next, 5 ml of GRP medium (Gibco) was added and the suspension was centrifuged at 1000 rpm for 5 mins. Obtained pellet of cells was resuspended in 10 ml of GRP medium with 10 mg/ml of DNase, incubated at 37 °C in humified atmosphere with 5% CO_2_ for 10 mins. Then, the pellet was triturated again and centrifuged at 1000 rpm for 5 mins, resuspended in 10 ml of GRP medium and plated on coated PLL/Laminin 25 ml flasks in 37 °C in humified incubator with 5% CO_2_. Cells were cultured for 5–10 days (1–2 passages) in GRP medium with bFGF, harvested with TrypLE Express (Gibco), cryopreserved in ATCC medium (LGC Standards, Teddington, UK), and stored in vapor phase liquid nitrogen until transplantation.

### *In vitro* characterization of GRP-hydrogel composites

To assess the survival and proliferation of GRPs after embedding in HA-based hydrogel – (Heprasil^®^, Tebu Bio, France) luciferase-expressing mouse glial restricted progenitors (msGRPs) were used. The msGRPs were isolated from light-producing transgenic mice as described previously^20^. The msGRPs (1 × 10^5^) were embedded in 100 µl of Heprasil (in a ratio 4:1 Heprasil to PEGDA Extralink^®^; Tebu Bio) and plated on 96-well plates. After 15–20 min when the hydrogel was fully solidified, the culture medium was added to the well. Monolayer cell culture without hydrogel was used as a control. Relative luminescence was measured daily, with addition of D-luciferin (Gold Biotechnology, St, Louis, USA) to the medium (15 µg/ml in culture media) and reading luminescent signal using Tecan plate reader (Männedorf, Switzerland).

### Animals

All animal experiments were approved by the University of Warmia and Mazury ethics committee, and were performed according to ARRIVE guidelines. Additionally for transplantation procedures on dogs written permission of dog owners were obtained.

#### Experimental animals (Pigs)

Eight juvenile, Large White domestic pigs (40 kg, both genders) were used. At least two weeks before transplantation animals were acclimated to the new environment and human presence to minimize stress associated with the experiment. Pigs had access to water and food *ad libitum*.

#### Experimental animals (Dogs)

Dogs were recruited for the study based on neurological exam identifying symptoms of DM and genetic test confirming mutations in the SOD-1 gene. For immunosuppression dogs received Equoral (cyclosporin, Teva Pharmaceuticals, Poland), in dose 100–200 mg per day, dependently on the weight of the animal (8–10 mg/kg), 1–2 months prior to transplantation. Cyclosporin concentration in blood was measured and maintained at 100–150 ng/ml. Immunosuppression was continued three months after transplantation to prevent rejection of the graft.

### Cells preparation

To optimize and study the safety of the methodology, more accessible BMMSC were used in transplantation in pigs. After demonstrating the safety of the transplantation in dogs suffering from DM, we have used cGRPs, having a potential therapeutic effect in the existing disease. Both cells types porcine bone marrow mesenchymal stem cells (pBMMSC) and canine glial restricted progenitors (cGRP) were prepared for the transplantation by the same way. One day before transplantation cells were thawed, plated and suspended in medium supplemented with 25 µg/ml SPION particles (Molday, BioPal, Worcester, MA, USA) for labeling and detection in MRI. Next day cells were washed with PBS, harvested and centrifuged at 1100 rpm for 5 min. Cells pellet was suspended in PBS or hydrogel (3 × 10^6^/ml; HyStem, Tebu Bio).

### Hydrogel preparation

The thiol-modified hyaluronic acid with thiol-modfied heparin (Heprasil^®^, HyStem^®^, Tebu Bio) was prepared in the same way in both experiments (cells transplantation in pigs and dogs) by dissolving in a phosphate buffered saline (Gibco) according to the manufacturer instructions with addition of 25 µg/ml SPION particles (Molday, BioPal) for increasing of the hipointensity signal in the MRI. Just before transplantation cells labelled with SPION were mixed with Heprasil® and 2 minutes prior to injection the hydrogel was cross-linked with Extralink^®^ - polyethylene glycol diacrylate (PEGDA) with the process of cross-linking slowly progressing over 20 min.

### MRI-guided intrathecal transplantation of porcine BMMSC

Eight juvenile Large White domestic pigs were divided into two groups. Pigs from group I were transplanted with pBMMSC in PBS, whereas pigs from group II were transplanted with pBMMSC embedded in HA- based hydrogel. During transplantation procedure animals were anesthetized with a combination of sevoflurane (2%) and propofol (3 mg/kg/h). Anesthetized pigs were placed under C-arm and x-ray guidance was used to navigate MRI-compatible catheter *via* lumbar puncture into thoracic section of the spinal cord. Animals were transported into MRI and positioned supine in a 3 T MRI scanner (Magnetom Trio, Siemens AG, Germany). Immediately before infusion SPIO-labeled cells were suspended in PBS and loaded into injection syringe directly (group I) or were suspended in hydrogel (group II). Intrathecal injection was initiated two minutes after mixing with a cross-linker and dynamic T2*-weighted images (GE-EPI TR/TE:1800/42 ms) were acquired to monitor biodistribution of cells in PBS/hydrogel in real-time. The MRI protocol also included T2 (TR/TE = 2000/117 ms) and T1 (TR/TE = 650/9.3 ms) scans prior and post cell delivery.

### MRI-guided intrathecal transplantation of canine GRPs

Five adult dogs (5–12 years) of various breeds (3 German Shepherds, 2 Hovawarts) suffering from degenerative myelopathy (DM) were subjected to the experiment. During the procedure animals were anesthetized with a combination of sevoflurane (2%) and propofol (3 mg/kg/h). The MRI-compatible catheter was introduced under x-ray C-arm guidance *via* lumbar puncture into thoracic section of the spinal cord. Then, dogs were transported into MRI and positioned supine in a 3 T MRI scanner (Magnetom Trio, Siemens AG). MRI protocol was as that for pig studies with T2*-weighted dynamic imaging of hydrogel biodistribution in real-time and T2,T1 scans prior and post cells delivery. Just after the procedure during recovery from anesthesia dogs were hydrated with Ringer’s fluid and were returned to their owners for recovery. The follow up monthly visits included blood analysis and neurological examination. MRI was done 1, 6 and 12 months post transplantation.

### Histopathological analysis

Spinal cords tissues of pigs were collected immediately after slaughter, 5 days after pBMMSCs transplantation, fixed in 4% paraformaldehyde, for 48 h at 4 °C, cryo-protected in 30% sucrose until sank, frozen on dry ice for 5 mins and kept at −80 °C for histological analysis. For dogs that succumb to the disease spinal cord was harvested and protected as above. Spinal cord tissue was cryo-sectioned at 20 μM using Hyrax C25 PLMC cryostat (Zeiss, Warsaw, Poland), transferred onto Superfrost slides (Menzel Gläser, Braunschweig, Germany) and processed for histological (HE, Prussian Blue) and immunofluorescent staining. For immunofluorescence, sections were dried in room temperature (RT) for 10 mins and washed 2 times in phosphate-buffered saline (PBS) for 10 min and in PBS (pH 7.45) containing 0.1% Triton X-100 (Sigma Aldrich) for 10 min followed by blocking in a PBS with 10% NGS (Normal Goat Serum; Sigma Aldrich) for 1 h at RT. Next, slides were incubated overnight at 4 °C with primary rabbit polyclonal antibodies diluted in PBS/10% NGS (rabbit anti IBA-1 1:50, rabbit anti GFAP 1:100; Abcam Cambridge, England; DAKO Santa Clara, CA, United States). The next day, sections were washed 3 times in PBS for 10 min, incubated with secondary antibody conjugated with fluorochrome Alexa 488 (Alexa Fluor 488 goat anti-rabbit IgG, 1:400; Thermo Fisher Scientific, Waltham, Massachusetts, United States) for 1.5 h at RT and washed in PBS. A negative control staining for the secondary antibody was performed by replacing the primary antibodies with 10% NGS (Sigma Aldrich). Microscopic sections were covered with DAPI mounting medium (Fluoroshield, Sigma Aldrich) and analyzed using a Zeiss Axio Observer microscope (Zeiss, Germany).

### *Ex vivo* MRI

For *ex vivo* MRI, spinal cords were placed in plastic container and imaged using a 3 T MRI scanner (Magnetom Trio, Siemens AG) using 15-channel surface coil. The MRI protocol included T2 transverse: ME2D (TR/TE = 1810/17 ms), TSE (TR/TE = 9230/107 ms) and PD (TR/TE = 4100/9.2 ms).

### Statistical analysis

Statistical analyses were performed using GraphPad Prism 5.0 (Graphpad Software, Inc, San Diego, CA). Statistical tests included one-way analysis of variance, followed by Dunnet’s *post hoc* test to determine the difference of final fractional signal decrease and epidural space whereas in relative immunofluorescence signal one-way analysis of variance was followed by Tukey’s *post hoc* test. All numerical data are presented as the mean ± standard error of the mean, and differences were considered as statistically significant at the 95% confidence level (P < 0.05).

## Data Availability

The datasets generated and analyzed during the current study are available from the corresponding author on reasonable request.
